# Development and practical validation of BUBBLESS tapping instrument for efficient bubble removal during antibody drug mixing

**DOI:** 10.1186/s40780-026-00539-5

**Published:** 2026-01-10

**Authors:** Masakazu Ozaki, Shoko Tanaka, Sakina Inoue, Toshiki Yoshii, Yuuko Nagatomi, Miwako Takasago, Naoto Okada, Takashi Kitahara

**Affiliations:** 1 Pharmacy Department, Yamaguchi University Hospital, 1-1-1, Minami-kogushi, Ube, Yamaguchi, 755–8505 Japan; 2 Department of Clinical Pharmacology, Yamaguchi University Graduate School of Medicine, 1-1-1, Minami-kogushi, Ube, Yamaguchi, 755–8505 Japan

**Keywords:** Mixing, Bubble removal, Syringe operation, Antibody drug, Aggregation

## Abstract

**Background:**

Antibody drugs play a central role in modern medical treatments, despite their tendency to aggregate under physical stimuli being a major challenge. Generically, these are prone to foaming, and bubble from syringes is typically manually removed by means of tapping. Hence, this study aimed to develop and evaluate the efficacy of a tapping instrument for bubble removal from syringes with minimal physical stress.

**Methods:**

The instrument was designed with a columnar shape to minimize contact area with the syringe surface. This instrument was named BUBBLESS, a coined term combining “bubble” and “less.” To measure the force applied to syringes, a cutting-force measurement system was employed. Twelve participants each drew 50 mL of distilled water into syringes and removed naturally occurring bubbles. Bubble removal was performed three times each by hand and using BUBBLESS per participant. Infliximab was the model antibody drug to evaluate aggregation, and its solutions were immediately imaged, following bubble removal by manual or BUBBLESS tapping, under a laser microscope.

**Results:**

A columnar shape, based on its contact area with the syringe, and polyacetal resin were used for developing BUBBLESS (weight: approximately 38.0 g). The median maximum force (interquartile range [IQR]) applied to the syringe was 129.1 (113.1–175.2) N by hand and 60.7 (47.9–80.9) N with BUBBLESS (*p* < 0.0001). The median total applied force (IQR) was 1,665,283 (1,176,238–2,445,051) N by hand and 575,061 (400,170.3–731,369.6) N with BUBBLESS (*p* < 0.0001). Laser microscopy revealed visible aggregation of infliximab following manual tapping but almost none after BUBBLESS tapping. No further changes were observed during a 10-min observation period in either case. The largest recorded horizontal and vertical diameters were approximately 12.5 and 13.4 μm, respectively, with no further changes observed in the aggregate during the 10-min observation period.

**Conclusions:**

This study shows the potential of the BUBBLESS tapping method as a novel approach to suppress antibody drug aggregation.

## Background

Presently, antibody drugs play a central role in modern medicine. However, their preparation as injectable medications presents a key challenge of drug aggregation under physical stimuli [1–3]. Reportedly, this aggregation reduces therapeutic efficacy and poses safety concerns such as immunogenicity [4–6]. Therefore, healthcare professionals commonly understand that strong physical stimuli should be avoided when administering antibody drugs. However, antibody drugs exhibit considerable proneness to foaming, with numerous bubbles frequently adhering to the inner surface of syringes during mixing. The only established method for removing these bubbles has been to apply physical stimuli to the syringe, which, in turn, may cause the antibody drug to aggregate upon excess stimuli. Presently, in addition to aggregation caused by physical stimuli during mixing, mechanical stress during post-manufacturing transportation has been reported to promote aggregation risk [7, 8]. These challenges highlight the need to develop a novel method that can remove bubbles from inside of syringes using weaker physical stimuli.

Traditionally, bubbles inside syringes have been removed manually by hand tapping, which often results in limited transmittance of force to the desired area, thereby requiring strong tapping. Moreover, variations in preparation techniques among individuals can lead to inconsistent application of physical stimuli to the syringe, with some individuals potentially applying excessive force. These inefficiencies are attributed to the softness of the hand and its broad contact area, which reduces energy transmission efficiency and necessitates greater applied force. We therefore hypothesized that a specially designed tapping instrument could efficiently transmit force to the syringe, enabling bubble removal with weaker physical stimuli. Despite various studies on bubble removal [9–12], none have approached the issue from this perspective. As the presence of bubbles in syringes can also cause volume measurement errors [10–12], establishing an effective and low-stress bubble removal method is of great importance.

Hence, this study aimed to develop a tapping instrument that allows bubble removal from syringes with reduced physical stimuli, along with evaluating its efficacy. Herein, the tapping instrument was designed and fabricated, the force applied to syringes were compared between hand and instrument, and aggregation under both manual and instrumental tapping was assessed using an antibody drug.

The findings of this study may provide an experimental basis for the establishment of a safe preparation protocol for antibody drugs that minimizes aggregation and removes bubbles within syringes even with weaker physical stimuli.

## Methods

### Materials

The syringes were manufactured by the TERUMO Corporation (Japan). Distilled water was purchased from Fuso Pharmaceutical Industries Ltd. (Japan). Infliximab was selected for antibody drug preparation to evaluate aggregation, as it has been reported to exhibit aggregation and self-association [13]. Infliximab BS for I.V. Infusion 100 mg NK was purchased from Nippon Kayaku Co. Ltd. (Japan). A cutting force measurement system (Kistler Instrumente AG, Switzerland) was used to measure the force applied to syringes. The DynoWare data acquisition system of type 5697 A, the laboratory charge amplifier of type 5080 A, the multicomponent dynamometer of type 9257B, and the DynoWare software of type 2825 A-02 were used in this study. A magnetic base, namely MB-T3 (KANETEC Co. Ltd., Japan), was used to fix the syringe to a multicomponent dynamometer. The aggregation of the antibody drug was observed under a laser microscope (OLS5100-EAF, Evident Corporation, Japan).

### Developing the tapping instrument

Various shapes, such as a baseball bat-like, ballpoint pen-like, and toothbrush-like, were considered, but we focused on designing an instrument whose center of gravity is aligned with the point of contact with the syringe. To achieve this goal, the volume and weight of the impact portion needed to be larger than the volume of the handle portion. Regarding the contact part, a columnar shape, baseball bat-like, was selected for the instrument to minimize contact area with the syringe surface. Following this, material fabrication was outsourced to an independent private manufacturer. The tapping instrument was produced using polyacetal resin (POM), which was selected owing to its weight, strength, and resistance to high-pressure steam sterilization and alcohol disinfection. A medical-grade POM material compliant with International Organization for Standardization (ISO) 10993, the global standard for medical device biocompatibility, was used. The developed tapping instrument was named BUBBLESS, a coined term combining “bubble” and “less.”

### Measurement of the force applied to the syringe

The measurement range for each channel of the laboratory charge amplifier in three-channel mode (X, Y, and Z) was set to a maximum of 300 N. The channel trigger was configured on the Y-axis, where the greatest force was applied (threshold: 0.3 volts [absolute value], pre-trigger: 10%). The total measurement duration was 10 s, with a sampling rate of 30,000 points per s (30,000 Hz).

A group of 12 participants performed the task of drawing 50 mL of distilled water into syringes, whereafter carefully removing any naturally occurring bubbles. Hereat, the syringes were fixed to the magnetic base and consistently positioned in the same orientation. The bubble removal criterion was defined as the level necessary to ensure accurate collection volume equivalent to that required in clinical mixing procedures. Each participant removed bubbles manually (direct tapping with a finger and a hand) and using BUBBLESS three times each.

### Laser microscopy of infliximab aggregation

Infliximab solution (10 mg/mL) was prepared per standard clinical procedures (gently injecting 10 mL of distilled water for injection per vial to dissolve, and then leaving to stand for 5 min). Thereafter, 10 mL of the solution was aspirated from the vial using a 50-mL syringe, and after removing the bubbles from this solution by hand or BUBBLESS tapping, the solutions were immediately imaged using a laser microscope. While the force measurement was conducted with statistically sufficient repetitions (*n* = 36), the microscopic observation in this study was performed as a preliminary qualitative assessment to visually confirm the potential impact of the reduced force. Therefore, the observation was conducted once for each condition. We have acknowledged this limitation in the Discussion section and emphasized the need for future robust validation with multiple lots. All observations were conducted within 5 min after tapping and continued for at least further 10 min. Simultaneously, the force applied to the syringes and the number of taps were standardized to the mean values using a cutting force measurement system (Table 1; *measured forces applied to syringes*).

**Table 1:** Number of taps, maximum force, and total force required for bubble removal

Participant	Hand			BUBBLESS		
	Number of taps	Maximum values (*N*)	Total values (*N*)	Number of taps	Maximum values (*N*)	Total values (*N*)
A	9	127.2	1,337,136	4	20.0	496,519
	3	138.9	1,157,057	8	44.7	793,103
	5	90.4	935,423	4	53.1	259,262
B	2	468.0	1,018,639	6	74.0	307,849
	6	495.8	2,328,718	2	84.7	396,919
	1	468.3	722,810	2	100.2	561,517
C	13	116.2	2,949,484	4	71.3	582,453
	16	169.8	1,911,289	3	87.9	646,628
	15	177.0	2,115,027	1	103.5	428,166
D	5	116.9	1,301,292	3	59.5	668,818
	6	141.0	1,077,919	2	57.5	298,494
	6	153.7	1,457,492	4	78.2	739,180
E	9	68.3	1,434,430	4	46.1	604,977
	13	73.6	2,516,096	10	69.4	1,003,614
	10	107.7	1,689,183	4	49.3	690,471
F	25	111.2	2,994,646	2	48.0	637,983
	9	119.4	1,233,782	1	28.2	531,274
	16	109.7	1,641,383	2	69.0	567,668
G	6	106.3	989,331	1	77.4	238,973
	13	122.6	1,965,955	1	81.7	200,378
	16	519.6	3,346,574	4	61.8	239,984
H	21	200.9	3,031,243	6	33.8	409,924
	9	196.0	1,759,517	12	58.2	707,939
	15	166.7	2,459,728	11	35.3	782,877
I	10	123.0	2,123,428	4	47.8	1,100,358
	15	112.0	2,401,022	3	48.7	809,838
	7	116.3	1,139,510	2	33.7	977,004
J	24	130.9	1,813,781	2	84.4	661,352
	20	159.5	2,531,154	10	77.5	1,069,694
	23	182.9	2,790,348	3	63.0	618,129
K	13	100.2	1,472,006	2	87.4	229,711
	3	124.7	1,098,763	4	92.3	511,086
	2	138.7	733,872	2	86.5	363,995
L	8	124.0	1,338,744	12	59.0	799,390
	15	186.4	2,675,368	10	54.2	564,245
	4	132.0	1,463,519	6	46.0	467,174
Mean	10.9	172.1	1,804,324	4.5	63.1	582,415
S.D.	6.5	117.7	726,088	3.3	30.0	240,706
Median	9.5	129.1	1,665,283	4	60.7	575,061
I.Q.R.	6–15	113.1–175.2	1,176,238–2,445,051	2–6	47.9–80.9	400,170.3–731,369.6

Abbreviations: S.D., standard deviation; I.Q.R., interquartile range

Initial imaging was conducted over a wide field using a 5× objective lens (MPLFLN 5×). The largest aggregate observed in each sample was then selected and imaged under 100× objective lens (LMPLFLN 100×). In both cases, the standard magnification was 21.6×, with the digital zoom set to 1×; hence, the total magnification achieved by the 5× and 100× objective lenses were 108× and 2,160×, respectively.

### Statistical analysis

Statistical analyses were conducted utilizing the JMP^®^Pro18 software. The Mann–Whitney U test was used to compare the number of taps, maximum force values, and total force values measured in the syringe. Statistical significance was set at *p* < 0.05.

## Results

### Developing the tapping instrument

**Fig. 1:**
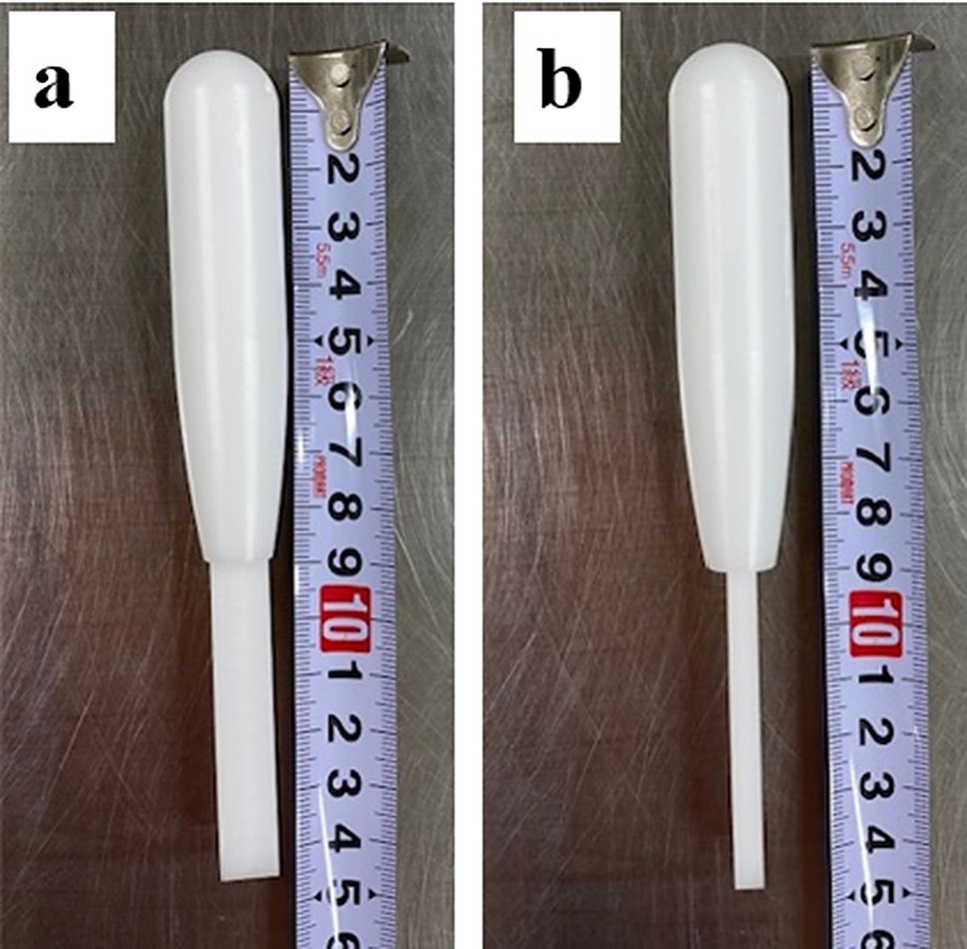
BUBBLESS tapping instrument. **a**: front image, **b**: side image. (The thick part is the impact part and the slim part is the handle)

Herein, five units of the BUBBLESS (Fig. 1) were developed successfully, weighing 37.79, 37.79, 37.07, 38.68, and 38.71 g (average weight: 38.0 g).

### Measurement of the force applied to the syringe

**Fig. 2:**
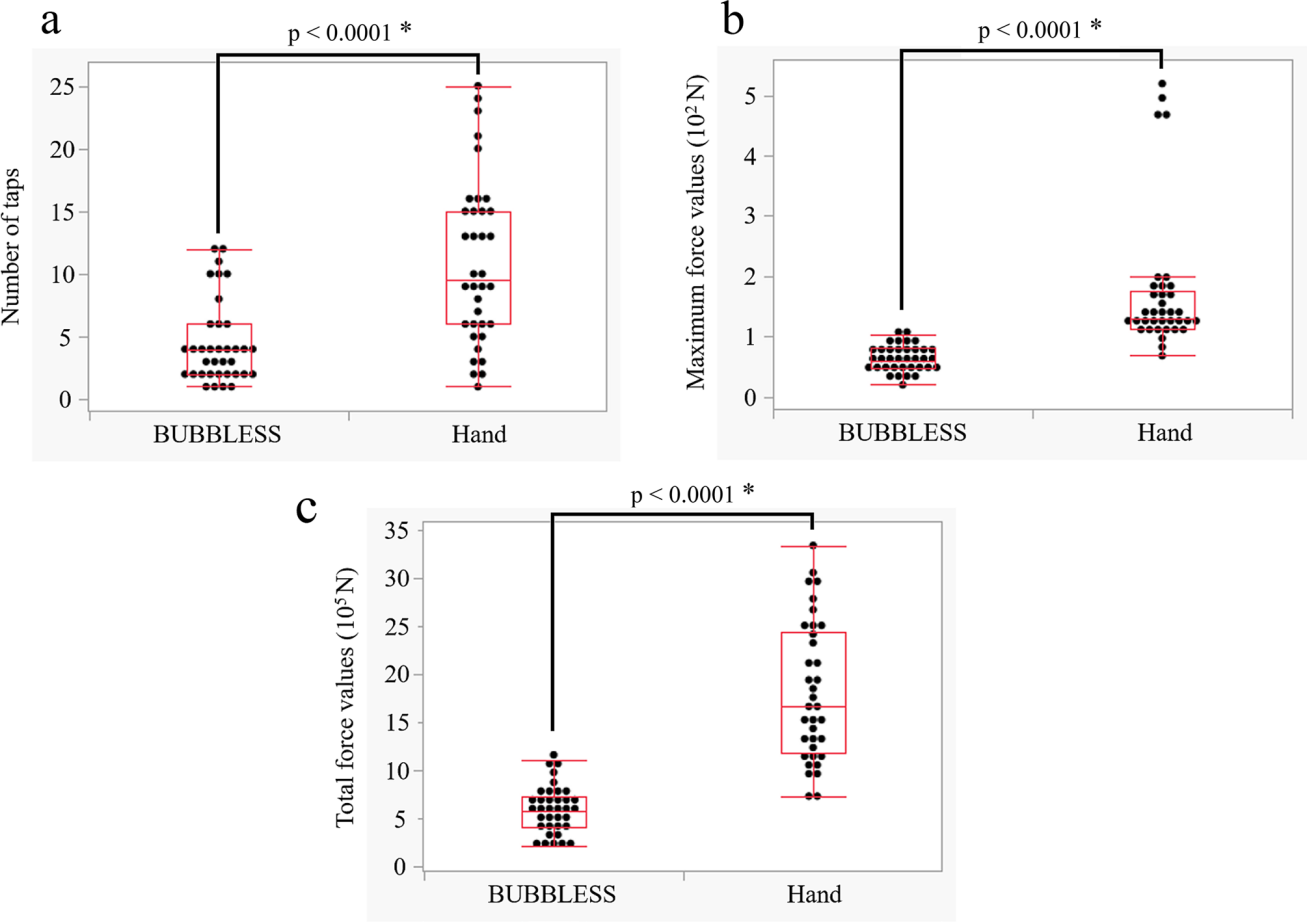
Boxplot for bubble removal by hand and BUBBLESS. **a**: number of taps, **b**: maximum force values, and **c**: total force values. * Mann–Whitney *U* test

Table 1 summarizes the number of manual and BUBBLESS tapping required for bubble removal, along with the maximum and total force (N) values applied to the syringe by the 12 participants. The median number of taps (interquartile range [IQR]) was 9.5 (6–15) and 4 [2–6] for manual and BUBBLESS tapping, respectively (*p* < 0.0001) (Fig. 2a). The median maximum force (IQR) applied to the syringe was 129.1 (113.1–175.2) N for manual and 60.7 (47.9–80.9) N for BUBBLESS tapping (*p* < 0.0001) (Fig. 2b). The median total force (IQR) was 1,665,283 (1,176,238–2,445,051) and 575,061 (400,170.3–731,369.6) N for manual and BUBBLESS tapping, respectively (*p* < 0.0001) (Fig. 2c).

### Laser microscopy of infliximab aggregation

**Fig. 3:**
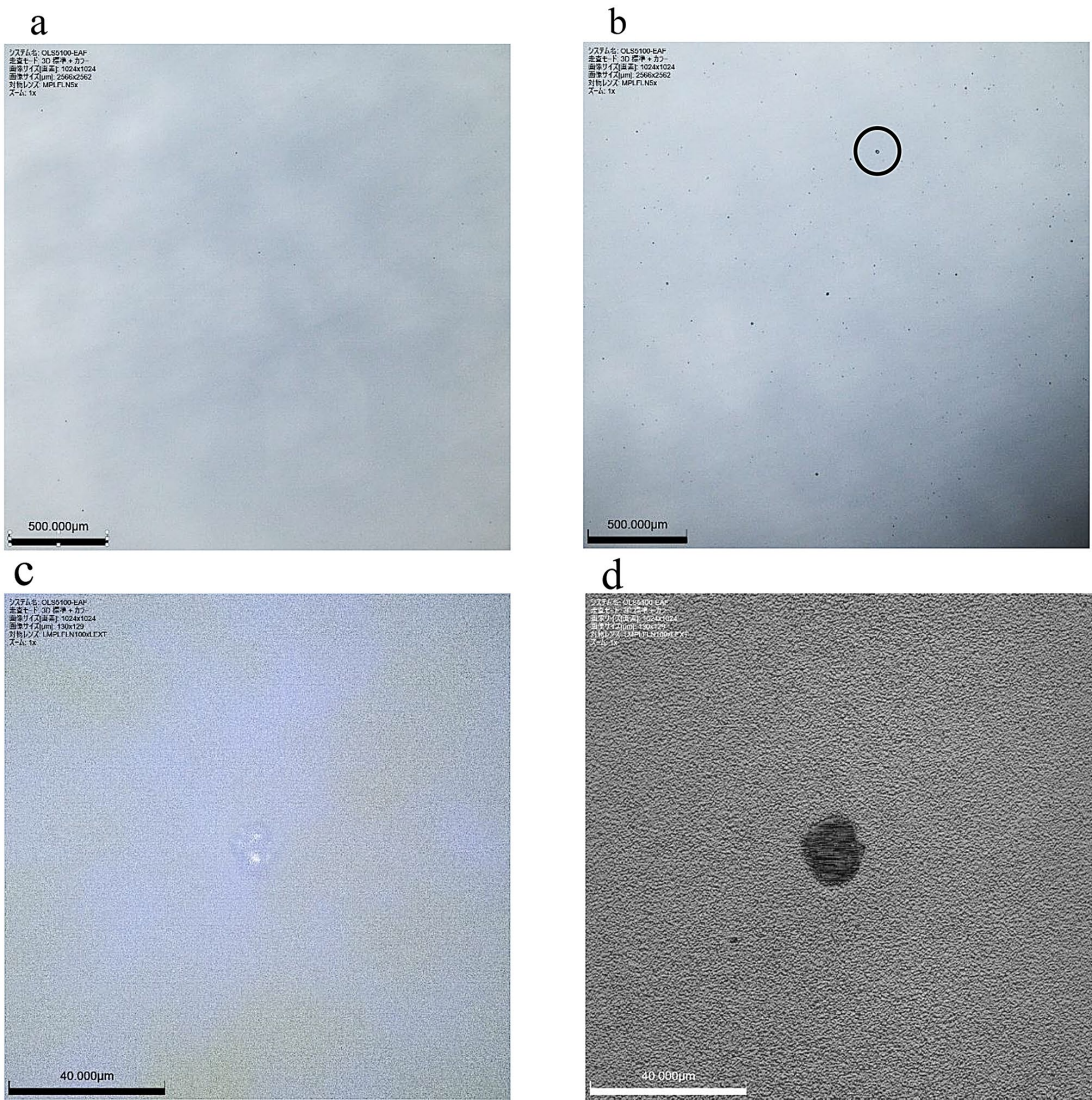
Laser microscopy images. **a**: Infliximab solution after BUBBLESS tapping, showing minimal aggregation, **b**: Infliximab solution after manual tapping, showing visible aggregates, (largest particle circled); scale bar: 500 μm, **c**: Color image of the circled particle under 100× objective lens (LMPLFLN 100x), **d**: Luminance image of the same particle under 100× objective lens (LMPLFLN 100x); scale bar: 40 μm

The images after tapping with BUBBLESS (Fig. 3a) and after manual tapping (Fig. 3b) obtained with the 5× objective lens (MPLFLN 5×) show that the particles were scarcely visible following BUBBLESS tapping but were clearly visible after manual tapping. In both cases, no morphological changes were detected during the 10-min observation period. The magnified view of the largest particle (Fig. 3c and d) imaged using the 100× objective lens (LMPLFLN 100×), along with its size profile analysis indicated an approximate horizontal diameter of 12.5 μm and a vertical diameter of 13.4 μm. No further structural changes were observed in this aggregate throughout the 10-min observation period.

## Discussion

In Japan, pharmacists routinely prepare various injectable drugs, including anticancer agents and high-calorie infusions, and bubble removal from syringes during their preparations can be influenced by the operator’s technical proficiency. However, from the standpoint of medication safety, drug preparation need to be performed safely and efficiently, independent of individual skill differences. Therefore, we developed a tapping instrument specifically designed to minimize technique-related variability, such as differences in the force applied to the syringe or the number of taps, and evaluated its practical effectiveness. We believe that such operational improvements are essential for advancing medical technology.

During development of BUBBLESS, the instrument was deliberately designed such that its center of gravity coincided with the point of contact with the syringe—at the center of its columnar shape—to achieve efficient force transfer. Furthermore, although target weight had not been set for the instrument, POM was selected as the material to make it as light as possible, and the average weight of the instrument was 38.0 g. While the weight of the instrument is a factor that influences bubble removal, this study did not perform a comparative analysis across different weights; this remains a limitation of the present research. Despite this limitation, the results demonstrated that BUBBLESS reduced individual variability in the total force applied to the syringe. Moreover, there was a substantial decrease in both the number of syringe taps and the maximum force applied, supporting the efficacy of the columnar design. Regarding the sample size of this experiment, 12 individuals participated three times, resulting in 36 data points for both manual tapping and BUBBLESS tapping, which we considered were sufficient for nonparametric tests comparing manual tapping and BUBBLESS tapping to confirm a significant difference [14]. Force measurements were recorded at 30,000 points per s, ensuring high accuracy. Tapping is a multivariate physical action influenced by not only magnitude but also velocity, impact angle, and consistency of contact position. In this study, to avoid differences in the performance of bubble removal methods among participants, other than the force applied, the syringes were fixed to the magnetic base and consistently positioned in the same orientation and the tapping angle was standardized. Thus, we ensured a reproducible experimental setup for each participant. The results revealed that some participants tapped syringes more than 20 times when removing bubbles by hand, with applied forces exhibiting wide variations (approximately 100–500 N). This variability reflects the physical burden associated with syringe preparation, which occurs repeatedly in daily operations. BUBBLESS was developed with the aim to reduce both physical strain and operator-induced variability. The bubble-removal tests were performed using 50-mL syringes. In clinical practice, antibody preparations may be handled in smaller syringes or occasionally in larger syringes. In this study, we measured the force generated when removing bubbles, but we believe that BUBBLESS exerts force without depending on the size of the syringe, if the syringe size is 10 mL or larger. If the syringe size is 10 mL or larger, the rate of incidence of insufficient tapping might be negligible. Conversely, excessive tapping, which could cause aggregation, might also not occur because tapping with BUBBLESS requires less force than tapping with hands. On the contrary, for syringes with narrow inner barrels, such as 1-mL syringes, the physical impact of BUBBLESS may not be optimized due to the small diameter. In such cases, minimizing air entry during aspiration remains the primary strategy, rather than post-aspiration tapping. In terms of sterilization, POM was used to fabricate the instrument; it is a medical-grade specification compliant with ISO that can withstand high-pressure steam sterilization. The material demonstrated no issues in sterile environments. However, ISO-compliant POM is relatively expensive, and future work should consider alternative materials to facilitate broader implementation of BUBBLESS.

Concerning the observation of antibody drug aggregation using infliximab, images captured after manual tapping revealed a clear increase in visible particles likely representing aggregates. This finding is consistent with previous studies indicating that antibody formulations tend to aggregate under physical stimuli [1–8]. During mixing, physical impact is often applied to remove bubbles, and the findings of this study confirm that such physical stimuli can induce aggregation. Reportedly, infliximab aggregation is reversible as time progresses [13], but in the present study, no changes were observed during the 10-min observation period. Therefore, the timeframe required for aggregate formation to be reversibly eliminated remain uncertain, along with their associated immunogenicity. In contrast, BUBBLESS tapping resulted in significantly fewer visible aggregates than those after manual tapping. These findings suggested that removing bubbles from infliximab formulation by BUBBLESS is more efficient than existing manual tapping methods. The infliximab formulation used for aggregation imaging consisted of a single vial, assuming no significant differences between manufacturing lots. Nevertheless, future studies should analyze multiple samples from multiple lots for robust validation. Furthermore, antibody preparations vary considerably in terms of physicochemical properties such as viscosity, stability, and aggregation tendency, and the use of only infliximab as the antibody preparation in this study is a limitation. It is necessary to verify whether similar results can be obtained for other antibody preparations. In addition, more studies are warranted for determining the threshold force required to induce aggregation utilizing measuring instruments such as a cutting force measurement system. Concurrently, characterizing particle size distributions throughout the solution using instruments such as laser diffraction particle size analyzers may provide more comprehensive insights into aggregation dynamics. The findings provide a research basis for similar studies on various antibody preparations in the future, with more data reflecting safer mixing practices.

## Conclusions

This study focused on developing and evaluating a novel tapping instrument for safer mixing of antibody drugs. Although validation with various antibody formulations is necessary, the developed tapping instrument, namely, BUBBLESS, may represent a novel tool to suppress antibody aggregation, thereby improving the quality of medical care.

## Data Availability

The datasets supporting the conclusions of this article are included within the article.

## Abbreviations

IQR: Interquartile range
ISO: International Organization for Standardization
POM: Polyacetal resins
